# Paraphlegia Cured by the Use of Magnetic Electricity

**Published:** 1860-09

**Authors:** J. Cerf

**Affiliations:** Wheeling, Ill.


### PARAPHLEGIA CURED BY THE USE OF MAGNETIC ELECTRICITY.

BY DR. J. CERF, OF WHEELING, ILL.

The cause of this severe malady is sometimes in the head, though more frequently in the spinal marrow. The symptoms of this form of palsy are as variable as the causes. With some, the disease commences with slight occasional pain referable to a part of the hip, thigh or leg, and frequently behind the protuberance at the head of the femur bone. Pain is not always present; fatigue frequently induces it, and rest relieves.—Weakness and pain now manifest themselves in the muscles at the lower part of the back, or in various parts of the legs. At times, the patient cannot walk, or even stand erect, without a paroxysm of pain following, which compels him to assume a recumbent position, as the only mode of obtaining partial relief. Locomotion is performed with difficulty; the legs threaten to give away, and bend under the patient at every step. After an interval of months or years, the legs become incapable of carrying the body beyond a few yards, and sometimes the erect position cannot be maintained, while the power of moving the legs may remain, and sensation be unaffected. Generally, a wasting of the paralysed limb is apparent, while sensation may be morbidly increased. On sudden and rapid changes of weather, the patient is attacked with painful neuralgic twitches, resembling those of Tic-Douloreux. The patient lives in this state an indefinite period.

*Case.*—Samuel Haegi, 17 years old, a robust looking young man, plethoric and strongly built, has suffered from an attack of paralysis. On the 15th of May, 1860, this young man was brought here to my office: it took 3 to 4 men to get him from the wagon. He could not walk without being assisted by somebody. His knees gave away and bent down at every step. Sensation was entirely gone, except by hard pressure. When I placed them in warm water, they showed bluish spots on different parts of his entire limbs. The lower and upper extremities were cold and trembling. He complained of no pains—was regular in his bowels, but not so with his urine, which was scanty. None of his family, ascending and descending, have been peculiarly disposed to hereditary disease.

*Treatment.*—I prescribed—

℞. Strychina, Gri.

Sulph. Acid. Arom., ʒ i.

Aquæ Destil., ℥ v.

Mix. 1 teaspoonful 3 times daily. Empl. Canth. 2-3 inches upon the Lumb. Vertebra, and Antim. Potass. Tart. ʒ i. Ung., Sempl., Mix. Ft., Ung., to be rubbed on the limbs every morning and evening, applying the Magnectic Electricity twice every day, and continue this treatment for two months in succession, with the exception, in place of the Antimon. oint. I use

℞. Delphinia, ℈ i.

Ung. Simpl., ℥ i.

Use as with the Antim. oint., and my patient has entirely recovered, and is now enjoying as good health as ever.

